# No relationships between frequencies of mind-wandering and perceptual rivalry

**DOI:** 10.1177/20416695231214888

**Published:** 2023-11-27

**Authors:** Souta Hidaka, Miyu Takeshima, Toshikazu Kawagoe

**Affiliations:** 13024 Rikkyo University, Niiza-shi, Japan; 13070 Sophia University, Chiyoda-ku, Japan; 13024 Rikkyo University, Niiza-shi, Japan; 13024 Rikkyo University, Niiza-shi, Japan; 212250 Tokai University, Kumamoto-shi, Japan

**Keywords:** mind-wandering, perceptual rivalry, online research, questionnaires, sustained attention to response task

## Abstract

Our minds frequently wander from a task at hand. This mind-wandering reflects fluctuations in our cognitive states. The phenomenon of perceptual rivalry, in which one of the mutually exclusive percepts automatically switches to an ambiguous sensory input, is also known as fluctuations in our perceptual states. There may be possible relationships between the mind-wandering and perceptual rivalry, given that physiological responses such as fluctuations in pupil diameter, which is an index of attentional/arousal states, are related to the occurrence of both phenomena. Here, we investigate possible relationships between mind-wandering and perceptual rivalry by combining experimental and questionnaire methods in an online research protocol. In Study 1, we found no statistically significant relationships between subjective mind-wandering tendencies measured by questionnaires and frequencies of perceptual rivalry for Necker-cube or structure-from-motion stimuli. Study 2 replicated the results of Study 1 and further confirmed no statistically significant relationships between behavioral measurements of mind-wandering tendencies estimated by sustained attention to response task and frequencies of perceptual rivalry. These findings suggest that mind-wandering and perceptual rivalry would be based on different mechanisms, possibly higher-level cognitive and lower-level perceptual ones.

Our minds frequently wander from a task at hand (perhaps from your current task reading this paper) in daily life. This mind-wandering (MW) is defined as the state in which attention and internal thoughts shift away from a to-be-focused task (Smallwood & Schooler, 2006). MW is reported to occur very frequently as 30–50% of our awaking time (Killingsworth & Gilbert, 2010) and be associated with both negative (e.g., stress) (Killingsworth & Gilbert, 2010; Mrazek et al., 2012, 2013) and positive (e.g., creativity) (Mooneyham & Schooler, 2013) mental states. Individuals’ MW tendencies have been measured not only by subjective questionnaire scoring (Carriere et al., 2013; Mrazek et al., 2013) but also by behavioral procedures such as experience sampling (Smallwood & Schooler, 2006).

Aside from the mind and thoughts, our percepts also fluctuate against sensory input (Blake & Logothetis, 2002). For instance, we can perceive either peoples’ face or the shape of a vase alternatively during the observation of the well-known Rubin's face-vase figure. This phenomenon is known as perceptual rivalry (PR). PR has been commonly demonstrated for a variety of stimuli, although different mechanisms may underlie stimuli with different properties, such as static and dynamic (Cao et al., 2018).

Intriguingly, MW and PR has been reported to be associated with fluctuations in the physiological index, pupil diameter. Pupil diameter is considered an index of the arousal/attentional state (Unsworth & Robison, 2016). Changes in pupil diameter reflect activation of the locus coeruleus (LC) in the brainstem, which releases a neurotransmitter, norepinephrine (NE), to a wide area of the brain (LC-NE system). The activities of the LC-NE system can modulate arousal/attentional levels and resulting cognitive performance (Aston-Jones & Cohen, 2005). Specifically, a lower activity of the LC-NE system is associated with a smaller pupil diameter (Unsworth & Robison, 2016). MW has been considered to reflect reduced attention to external input and shift of attention towards internal thoughts (Schooler et al., 2011). Consistent with this idea, pupil diameter is reported to become smaller just before and during the occurrence of MW (Grandchamp et al., 2014; Unsworth & Robison, 2016, 2018; but see also Pelagatti et al., 2020). PR is also demonstrated to be related to changes in pupil diameter (Einhäuser et al., 2008): pupil diameter dilates just before and during the occurrence of PR commonly for various types of static and dynamic ambiguous sensory inputs. Dilation of pupil diameter was also shown to be associated with sustained perception, namely less PR. These findings suggest that both MW and PR are commonly associated with changes in arousal/attentional levels.

Given that there may be a common mechanism of arousal/attentional processes underlying fluctuations in MW and PR, and that perceptual processes such as perceptual decoupling with reduced responsibility for incoming stimuli are considered to be involved in MW (Baird et al., 2014; Schooler et al., 2011), there may be possible relationships between fluctuations in cognitive (MW) and perceptual (PR) states. To the best of our knowledge, however, no study has investigated this possibility. The current study exploratory examined the potential relationship between MW and PR. We performed two studies (Studies 1 and 2) by combining experimental and questionnaire methods in an online research protocol. In both studies, we estimated an individual's MW tendencies using the Mind-Wandering Questionnaire (MWQ) (Mrazek et al., 2013). Since it has been pointed out that MW consists of different components with or without people's intentions (Seli et al., 2016b), we also used Deliberate (MW-D) and Spontaneous (MW-S) Mind-Wandering scales (Carriere et al., 2013) as measurements. We also assessed individuals’ PR tendencies for different types of stimuli, static (Necker cube: NC) and dynamic (structure-from-motion: SfM) ambiguous stimuli (Figure 1A). Study 2 further introduced an experience-sampling method as a behavioral measure of MW. A thought probe was presented during a sustained attention to response task (SART) in which participants were asked to complete a Go/No-go task with a low frequency of a no-go cue. We assessed participants’ MW tendencies distinguished from the other mind's states, such as task-related thoughts (TRTs) (Smallwood et al., 2004; Stawarczyk et al., 2012) or thinking nothing at all (mind blanking; MB) (Kawagoe et al., 2019; Ward & Wegner, 2013). The number of correct responses and reaction times (RTs) were also used as behavioral indices.

**Figure 1.**
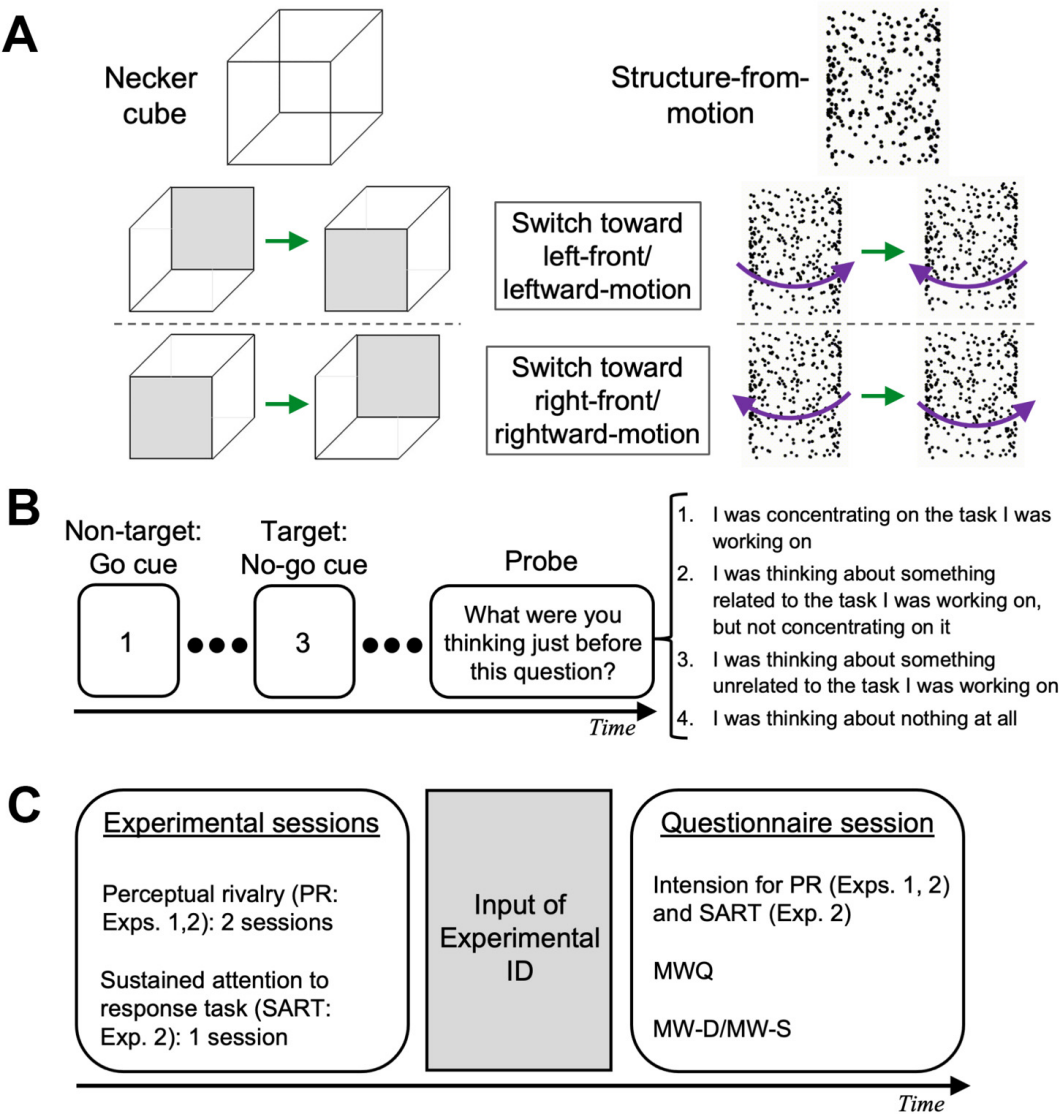
(A) Necker cube and structure-from-motion stimuli and schematic illustrations of perceptual rivalry. (B) Temporal sequence of events for the sustained attention to response task. (C) Temporal sequence of Studies 1 and 2.

Based on the previous findings on the relationships between changes in pupil diameter and MW (Grandchamp et al., 2014; Unsworth & Robison, 2016, 2018) and PR (Einhäuser et al., 2008), we predicted that higher frequencies of PR would be associated with stronger subjective and behavioral tendencies of MW. This idea also seemed to be consistent with the findings that older people tend to show fewer MW (Maillet & Schacter, 2016; Maillet et al., 2018) and more stable (less) PR during passive observations of ambiguous stimuli (Aydin et al., 2013; Cao et al., 2018). These age-related effects and positive relationships among the subjective indices of MW (Chiorri & Vannucci, 2019; Ostojic-Aitkens et al., 2019; Seli et al., 2015) and between the subjective and behavioral indices of MW (Kajimura & Nomura, 2016; Kawagoe et al., 2020; Mrazek et al., 2013; Yamaoka & Yukawa, 2019) were also expected to appear.

## Methods

### Participants

In study 1, we recruited participants using the database and platform of an online survey company (iBRIDGE Corporation, Japan). From January 15–16, 2021 we performed a screening for 1000 participants, equally consisting of people from the following age groups: 20s, 30s, 40s, and 50s. Gender was equally distributed across the age groups. We extracted 480 participants (197 females, mean (SD) age was 41.86 (10.78) years), who answered that they could participate in the later main study using a PC and Google Chrome. In the main study, 301 of the prescreened participants participated from January 19-February 1, 2021. We excluded 120 participants for the questionnaires, 173 for NC, and 165 for SfM from the analyses (see the “Data exclusion” section for details). A total of 181 (72 females; mean (SD) age: 42.96 (10.00) years) participants’ data were analyzed for questionnaires data. Among those, 128 (49 females; 43.59 (9.71) years) and 136 (55 females; 43.01 (9.81) years) participants’ data were used for the analyses regarding NC and SfM, respectively.

In Study 2, we recruited volunteers from authors’ acquaintances and affiliations because of the low completion rate and high exclusion rate for the participants recruited by the online survey company in Study 1. We collected data from 133 participants using the Google Forms platform from March 8, to September 24, 2021. We excluded 33, 38, 40, and 50 participants for questionnaires, NC, SfM, and SART from the analyses, respectively (see the “Data exclusion” section for details). Consequently, 100 (70 females; mean (SD) age: 25.12 (10.98) years) participants’ data were analyzed for questionnaires. Among those, 95 (68 females; 25.29 (11.24) years), 93 (68 females; 24.82 (10.82) years), and 83 (60 females; 24.70 (10.29) years) participants’ data were used for the analyses regarding NC, SfM, and SART, respectively.

Before data collection, we performed a power analysis for multiple linear regression, using G*Power 3.1 software (Faul et al., 2009) with an effect size of *f*^2^  =  0.15, alpha of 0.05, power of 0.8, and three predictors. This indicates that 77 participants were required. Our sample size was appropriately powered to detect a comparably sized effect.

All procedures were approved by the Ethics Committee of the Department of Psychology, Rikkyo University (reference number: 20-67), and were performed in accordance with the approved guidelines and the Declaration of Helsinki. Informed consent was obtained from each participant prior to participation. Our data and analysis code have been made publicly available via the Open Science Framework and can be accessed at https://osf.io/sx96j/.

### Procedures

At the beginning of each study, we gave the following instructions to the participants: “This study survey is performed to investigate how individuals’ thoughts and percepts are related. You will be asked questions about your daily thinking patterns. You will be asked to perform a simple experiment on how the images look. Please follow the instructions on the experimental webpage. Your task is very simple and will take about 5 (Study 1) /15 (Study 2) minutes.” Each study started with experimental sessions, and the participants were asked to complete the questionnaires later (Figure 1C).

#### Experimental Sessions

We ran the PR experimental session as an online experiment programed and controlled it with lab.js (Henninger et al., 2021). We presented NC or SfM stimuli (Figure 1A) with a 300  ×  300 pixel image size against a white (255 gray-scale number) background made by Matlab (MathWorks, Natick, MA) with the Psychophysics Toolbox (Brainard, 1997; Pelli, 1997). The NC stimulus consisted of two square frames drawn with 200-pixel black (0 gray scale number) lines. The square frames were separated by 100 pixels in both the vertical and horizontal directions and were connected to four other black lines. The SfM stimulus consisted of 200 dots of 7 pixels randomly located within the cylinder window with a height of 280 pixels and a width of 200 pixels. Each dot moved 100°/s at 30 Hz. During the 90 s’ presentation, one of these stimuli appeared at the center of the screen. Participants were asked to respond by pressing a key: they pressed the “f” key when their perception switched to left-front/leftward-motion and the “j” key when their perception switched to right-front/rightward-motion during the observations of NC/SfM stimuli. We provided detailed instructions regarding the possible perception of stimuli and how responses were made before starting the experimental session. The presentation order of the stimuli was randomized among participants. After the experimental session, a unique experimental ID was issued. Participants were asked to write it down and input it afterward, which enabled us to identify individual data among the sessions.

In Study 2, we also introduced SART **(**Figure 1B) as a Go/No-go task using jsPsych (de Leeuw, 2015). During the task, a single number from one to nine was randomly displayed at the center of the screen. The participants were asked to press the space key as soon as possible when a number (nontarget) other than “3” was presented (Go task). If “3” (target) was displayed, they were asked not to push the key and to keep waiting until the next number appeared (No-go task). Each number lasted for 250 ms at 1350 ms intervals. The experiment consisted of 25 consecutive blocks without rest, and the numbers were presented 20 times per block (500 trials in total). In each block, the target appeared in 10% of the trials (50 times in total). We also presented a probe during SART with the following question and alternatives: “What were you thinking just before this question? 1: I was concentrating on the task I was working on (Focused), 2: I was thinking about something related to the task I was working on, but not concentrating on it (TRT), 3: I was thinking about something unrelated to the task I was working on (MW), and 4: I was thinking about nothing at all (MB)”. Participants were asked to choose one of these alternatives. The probes were presented seven times at the end of one of the SART blocks. The probes appeared randomly among 25 SART blocks so that participants could not expect the presentation timing of the probes. We first introduced SART and then PR because we assumed that many participants would abort their participation if the reversed order was assigned because of the heavier task load of SART especially in the online research protocol.

We asked the participants to complete the experimental sessions using a PC with Google Chrome, which was the most compatible with our online research protocol. The experimental programs were run on a domestic server at Rikkyo University.

#### Questionnaires

Immediately after the experimental session, the participants were required to input their unique experiment ID. They were then asked to evaluate how they intentionally tried to induce PR with a 7-point Likert scale ranging from 1 (strongly disagree) to 7 (strongly agree) with a middle point 4 (neutral). In Study 2, we also asked to evaluate how they intentionally tried to wander their minds during the SART task using a 7-point Likert scale.

We measured general MW tendencies using the Japanese version (Kajimura & Nomura, 2016) of the MWQ (Mrazek et al., 2013). The MWQ consists of 5 items with a 6-point Likert scale ranging from 1 (almost never) to 6 (almost always). We also measured the deliberate and spontaneous aspects of MW using the Japanese version (Yamaoka & Yukawa, 2019) of MW-D/MW-S (Carriere et al., 2013). MW-D/MW-S consisted of eight items, four items for each questionnaire, with a 7-point Likert scale ranging from 1 (rarely/not at all true) to 7 (a lot/very true). The presentation order of the MWQ and MW-D/MW-S was randomized among the participants.

We included the item “please check the left- and right-most answer in this question” as filler questions to detect the use of an inappropriate (“Satisfice,” e.g., the strategy to fill the left-/right-most marks for all items without reading them) strategy (Krosnick, 1991) in the midst location of each MWQ and MW-D/MW-S, respectively. For demographic information, we also collected participants’ ages and genders.

### Data Analyses

Data analyses were performed using R software (version 4.0.3) (R Core Team, 2020) with psych (Revelle, 2017), stringer (Wickham, 2019), rapportools (Blagotić & Daróczi, 2021) and rstatix (Kassambara, 2020) packages and JASP (JASP Team, 2023). The alpha level for statistical tests was set at 0.05 with the Bonferroni correction.

#### Questionnaires

We calculated the sum of five items’ scores for MWQ and that of four items scores for MW-D and MW-S. We also used 1–7 points scores for the intention of PR and SART.

#### Behavioral Measurements

For PR, we counted the number of the pressed key changes (i.e., reported perceptual switching) for 90 s stimulus presentation as an index of PR for NC and SfM. As for SART in Study 2, the percentages of correct responses for the targets and nontargets, the mean and standard deviation of RT for the nontargets, and the sum of each response category (Focused, TRT, MW, or MB) for the probes were calculated.

#### Data Exclusion

Firstly, we excluded the data of participants whose responses to the filler question were wrong so that their strategy was regarded as inappropriate (“Satisfice”) (60 and 1 in Studies 1 and 2, respectively). We also excluded participants whose experimental ID was inappropriate (10 in Study 1 and 17 in the PR task and 29 in the SART in Study 2). For the PR task, data were excluded if no key presses were made (i.e., incompletion/abortion of task) for either NC or SfM stimuli (39 and 14 in Studies 1 and 2, respectively) and if there were no response changes (i.e., no occurrence of PR) for both stimuli (11 and 1 in Studies 1 and 2, respectively). We excluded the data if the deviation of the PR stimuli duration was over 1 s for each NC and SfM stimulus (14 and 8 in Study 1; 1 for each in Study 2). Based on visual inspection of distributions with the assumption that the number of key presses and that of the pressed key changes should be positively correlated, we excluded the data where the difference between the number of responses and that of the pressed key changes was over 11 for each NC and SfM stimulus (32 and 31 in Study 1; 1 and 3 in Study 2). Based on visual inspection of distributions, we further excluded the data where the number of responses or changes in responses exceeded 2 SD for each NC and SfM stimulus (7 and 6 in Study 1; 3 for each in Study 2). As for SART, we excluded three participants whose response error rate for the nontarget was over 0.5, and two participants whose standard deviation of RT for the nontarget was over 300 ms. We confirmed that the duration of stimulus presentation and blank period was stable in SART, where the maximum deviations were less than 100 ms.

#### Statistical Analyses

Since all indices in Study 1 (Shapiro-Wilk test: *Ws* < 0.98, *ps* < .003) and almost all indices in Study 2 (*W*s < 0.97, *p*s < .02) were not normally distributed, we used a nonparametric method for correlation analyses (Kendall's τ) and comparisons (Mann–Whitney *U* test). We also performed multiple linear regression analyses with Gaussian distribution. We checked the normality of the residuals based on the *Q*-*Q* plots (Supplemental Figure S1). We reported Bayes factors for these analyses as estimations of the extent to which the null or alternative hypothesis was supported with the default setting of JASP, considering more than 3 or less than 1/3 of the Bayes factors as a criterion.

## Results

### Study 1

First, we investigated whether the measurements of MW or PR were correlated with each other. As for the measurements of MW, there were significant positive correlations among MWQ, MW-D, and MW-S (*τs* > 0.24, *ps* < .001, BFs_10_ > 5.56  ×  10^3; Table 1, Supplemental Figure S2A). The number of PRs was not significantly correlated between NC and SfM (*τ*  =  −.02, *p*  *=*  .78, BF_10_  =  0.13; Supplemental Figure S2B). The subjective intention for PR was not significantly correlated with the number of PRs for NC (*τ*  =  −0.07, *p*  =  .31, BF_10_  =  0.22) and SfM (*τ*  =  0.12, *p*  =  .06, BF_10_  = 0.99), while the null hypothesis was not clearly supported by Bayes factors for SfM.

**Table 1.** Correlations, *P*-values, and Bayes factors among MWQ, MW-D, and MW-S scores in Study 1.

	τ	*P*	BF_10_
MWQ-MW-D	**0**.**24**	**<.001**	5.56 × 10^3
MWQ-MW-S	**0**.**49**	**<.001**	6.72 × 10^19
MW-D-MW-S	**0**.**44**	**<.001**	6.45 × 10^15

Bold letters indicate statistical significance with corrected *p* values. MWQ = Mind-Wandering Questionnaire; MW-D = Mind-Wandering Deliberate; MW-S = Mind-Wandering Spontaneous.

We also investigated the relationships between measurements of MW or PR and the demographic measurements. We found significant negative correlations between age and MW tendencies (*τs* > −0.21, *ps* < .001, BFs_10_ > 3.98  ×  10^2) except for MWQ (*τ*  =  −0.10, *p*  =  .06, BF_10_  =  0.73), but not for PR (*τs* < 0.04, *ps* > .52, BFs_10_ < 0.14) (Table 2; Supplemental Figure S2C). There were no significant differences in MW tendencies and the number of PRs between genders, while the null hypothesis was not clearly supported by Bayes factors for NC (*ps* > .06, BFs_10_ < 0.87; Table 3).

**Table 2.** Correlations, *p*-values, and Bayes factors between age and MW questionnaires’ scores or the numbers of PR in Study 1.

Age	τ	*P*	BF_10_
MWQ	−0.10	.06	0.73
**MW-D**	**−0**.**21**	**<.001**	3.98 × 10^2
**MW-S**	**−0**.**23**	**<.001**	4.69 × 10^3
NC	−0.04	.52	0.14
SfM	0.00	.95	0.11

Bold letters indicate statistical significance with corrected *p* values. MWQ = Mind-Wandering Questionnaire; MWD = Mind-Wandering Deliberate; MW-S = Mind-Wandering Spontaneous; NC = Necker cube; SfM = structure-from-motion.

**Table 3.** Results of Mann–Whitney *U*-tests with *p*-values and Bayes factors between gender regarding MW questionnaires’ scores or the numbers of PR in Study 1.

Gender	*U*	*P*	BF_10_
MWQ	3862.5	.86	0.17
MW-D	4233.5	.37	0.28
MW-S	4246.0	.35	0.18
NC	2503.0	.06	0.87
SfM	1558.0	.22	0.28

MWQ = Mind-Wandering Questionnaire; MW-D = Mind-Wandering Deliberate; MW-S = Mind-Wandering Spontaneous; NC = Necker cube; SfM = structure-from-motion

Our main purpose was to investigate the possible relationships between the MW and PR. We performed a multiple linear regression analysis with the MWQ, MW-D, and MW-S scores as explanatory variables and the number of PRs for either NC or SfM as a dependent variable. We found no significant model-fit for each model (NC: *F*(3, 124)  =  0.74, *p*  =  0.53, VIFs < 2.29, BF_10_  =  0.05; SfM: *F*(3, 132)  =  0.30, *p*  =  0.83, VIFs < 2.35, BF_10_  =  0.08).

### Study 2

We performed Study 2 to check the reproducibility of the findings in Study 1. We also tested whether the behavioral measurement of MW in SART could be related to the number of PRs.

Firstly, we investigated the relationships for the measurements of MW. There were significant positive correlations among the subjective measurements of MW (*τs* > 0.18, *ps* < .01, BFs_10_ > 4.56; Table 4, Supplemental Figure S3A). Regarding SART (Table 5), the number of MW responses for the probes was not significantly correlated with neither the mean or SD of RT for the nontarget nor the error rates for the target (*τs* < 0.14, *ps* > .11, BFs_10_ < 0.76). The error rates for the target were significantly correlated with the mean RT (*τ*  *=*  −0.41, *p* < .001, BF_10_  =  3.90  ×  10^5; Supplemental Figure S3B) but not with that of SD (*τ*  *=*  −0.16, *p*  =  .04, BF_10_  =  1.25). The subjective intention for MW during SART was significantly correlated with the number of MW responses for the probe (*τ*  =  0.28, *p*  =  .002, BF_10_  =  1.66  ×  10^2) and the error rates for the target (*τ*  =  0.27, *p*  =  .001, BF_10_  =  94.28), but not with the mean (*τ*  =  0.02, *p*  =  .81, BF_10_  =  0.15) and SD (*τ*  =  0.10, *p*  =  .23, BF_10_  =  0.33) of the RT for the nontarget. As for the relationships between the subjective and behavioral measurements of MW, we found significant positive relationships between MWS and the error rates for the target (*τ*  =  .24, *p*  *=*  .002, BF_10_  =  23.50; Table 6, Supplemental Figure S3C). The negative relationship between MWS and the mean of RT (*τ*  =  −.21, *p*  *=*  .005, BF_10_  =  7.84) and the positive relationship between MWQ and the error rates for the target (*τ*  =  .20, *p*  *=*  .01, BF_10_  =  4.59) were also supported by the alternative hypothesis with Bayes factors, although the corrected p-values did not reach the significance levels. The relationships for the other pairs were not statistically significant (*τs* < 0.16, *ps* *>* .07, BFs_10_ < 1.20).

**Table 4.** Correlations, *p*-values, and Bayes factors among MWQ, MW-D, and MW-S scores in Study 2.

	τ	*P*	BF_10_
MWQ-MW-D	**0**.**19**	**.01**	5.55
MWQ-MW-S	**0**.**45**	**<.001**	2.18 × 10^8
MW-D-MW-S	**0**.**18**	**.01**	4.56

Bold letters indicate statistical significance with corrected *p* values. MWQ = Mind-Wandering Questionnaire; MW-D = Mind-Wandering Deliberate; MW-S = Mind-Wandering Spontaneous.

**Table 5.** Correlations, *p*-values, and Bayes factors among the indices of SART in Study 2.

	τ	*P*	BF_10_
MW probe-RT mean	−0.09	.31	0.27
MW probe-RT SD	−0.06	.51	0.19
MW probe-error rate	0.14	.11	0.76
**Error rate-RT mean**	**−0**.**41**	**<.001**	3.90 × 10^5
Error rate-RT SD	−0.16	.04	1.25

Bold letters indicate statistical significance with corrected p values. SART = sustained attention to response task; RT = reaction time; MW = mind-wandering.

**Table 6.** Correlations, *p*-values, and Bayes factors of the indices of MW among the questionnaires and SART in Study 2.

		MWQ	MWD	MWS
MW probe	τ	0.07	0.05	0.16
	*P*	.39	.61	.07
	BF_10_	0.23	0.17	1.20
RT mean	τ	−0.12	0.05	−0.21
	*P*	.12	.55	.005
	BF_10_	0.51	0.17	7.84
RT SD	τ	0.08	0.15	−0.04
	*P*	.29	.06	.57
	BF_10_	0.26	0.91	0.17
Error rate	τ	0.20	0.14	**0** **.** **24**
	*P*	.01	.07	.**002**
	BF_10_	4.59	0.82	23.50

Bold letters indicate statistical significance with corrected *p* values. MWQ = Mind-Wandering Questionnaire; MW = mind-wandering; SART = sustained attention to response task; RT = reaction time.

Regarding the relationships for the measurements of PR, the number of PRs was positively correlated between NC and SfM, while the alternative hypothesis was not clearly supported by Bayes factors (*τ*  =  0.16, *p*  *=*  .04, BF_10_  =  1.49; Supplemental Figure S3D). The subjective intention for PR was not significantly correlated with the number of PRs neither for NC (*τ*  =  0.08, *p*  =  .32, BF_10_  =  0.24) or SfM (*τ*  =  −0.08, *p*  =  .33, BF_10_  =  0.24).

For the demographic measurements, there were no significant differences in MW tendencies and the number of PR between genders, while the null hypothesis was not clearly supported by Bayes factors for the MW-S score (*ps* > .03, BFs_10_ < 0.86; Table 7). Since most participants ages were 20s in Study 2, we did not perform correlation analyses regarding age.

**Table 7.** Results of Mann–Whitney *U*-tests with *p*-values and Bayes factors between gender regarding MW questionnaires’ scores, the indices of SART, or the numbers of PR in Study 2.

Gender	*U*	*P*	BF_10_
MWQ	1157.5	.42	0.30
MW-D	1160.0	.41	0.26
MW-S	1335.5	.03	0.86
MW probe	659.5	.75	0.26
RT mean	665.0	.80	0.27
RT SD	653.0	.71	0.24
Error rate	602.0	.37	0.30
NC	874.0	.72	0.26
SfM	913.5	.72	0.25

MWQ = Mind-Wandering Questionnaire; MW-D = Mind-Wandering Deliberate; MW-S = Mind-Wandering Spontaneous; NC = Necker cube; SfM = structure-from-motion; SART = sustained attention to response task; RT = reaction time.

Regarding our main analyses to investigate the relationships between the MW and PR, the multiple linear regression analysis with the MWQ, MW-D, and MW-S scores as explanatory variables showed no significant model-fit for PR (NC: *F*(3, 91)  =  0.29, *p*  =  .83, VIFs < 1.82, BF_10_  =  0.04; SfM: *F*(3, 89)  =  0.35, *p*  =  .79, VIFs < 1.84, BF_10_  =  0.04). For the SART, we used the mean RT for the nontarget and the error rates for the target as well as the number of MW responses for the probes as explanatory variables based on the results of the correlation analyses. We found no significant model-fit (NC: *F*(3, 77)  =  0.60, *p*  =  .62, VIFs < 1.50, BF_10_  =  0.07; SfM: *F*(3, 74)  =  0.52, *p*  =  .97, VIFs < 1.45, BF_10_  =  0.06).

## Discussions

This study exploratory investigated the possible relationship between MW and PR. The results of Studies 1 and 2 did not find statistically significant relationships between the subjective and behavioral indices of MW and the number of PR.

As for the measurements of MW, our results showed some consistency with previous findings. We found a positive relationship between MWQ and MW-D/S as well as MW-D and MW-S (Chiorri & Vannucci, 2019; Ostojic-Aitkens et al., 2019; Seli et al., 2015) in both studies. Age-related negative relationships were also observed between the subjective MW tendencies measured by the questionnaires in Study 1 (Maillet & Schacter, 2016; Maillet et al., 2018). We further observed a statistically significant relationship between the subjective (MW-S) and behavioral (the error rates for the target in SART) indices of MW (Kajimura & Nomura, 2016; Kawagoe et al., 2020; Mrazek et al., 2013; Yamaoka & Yukawa, 2019) in Study 2, indicating a positive relation for unintentional subjective and behavioral tendencies of MW. Additionally, a statistically significant relationship between participants’ intention for MW and behavioral indices of MW was observed in Study 2: the subjective intention score for MW was significantly correlated with some indices for SART (MW response for probe and the error rates for the target) (Seli et al., 2016a, 2016b). These findings suggest that our data reliably estimated the individuals’ MW tendencies.

We used traditional NC and SfM PR stimuli in the current study. Over 90% of our participants reported at least one PR for both stimuli (181/192 and 100/101 data in Studies 1 and 2, respectively). We found no statistically significant and significant but weak positive correlations between NC and SfM PR stimuli in Studies 1 and 2, respectively. These findings seem to be consistent with previous findings suggesting the involvements of independent mechanisms for PR stimuli with different properties (i.e., static and dynamic) (Cao et al., 2018). Notably, the subjective intention score for PR was not significantly correlated with the behavioral indices of PR. Based on these results, we could assume that our procedures reliably measured the tendencies of perceptual alternations for ambiguous stimuli.

Based on the previous findings showing the involvement of changes in pupil diameter, which is an index of arousal/attentional state and resulting cognitive performance (Aston-Jones & Cohen, 2005; Unsworth & Robison, 2016), for MW (Grandchamp et al., 2014; Unsworth & Robison, 2016, 2018) and PR (Einhäuser et al., 2008), we investigated potential associations between MW and PR. In Study 1, we found no statistically significant relationships between the subjective measurements of MW and the frequencies of PR. We replicated this result in Study 2. We further found no statistically significant relationships between the behavioral measurements of MW and the frequencies of PR.

Our findings suggest that MW and PR are based on distinct psychological and neural mechanisms, although it has been suggested that perceptual processes such as perceptual decoupling with reduced responsibility for incoming stimuli are involved in MW (Baird et al., 2014; Schooler et al., 2011).

MW is assumed to be related to cognitive processes such as attention (Schooler et al., 2011), internal thoughts (Smallwood & Schooler, 2006), intension (Seli et al., 2016a, 2016b), and metacognition (Kawagoe & Kase, 2020). In addition, MW tendencies have been reported to be modulated by task loads (Forster & Lavie, 2009; Seli et al., 2016a) and working memory capacity (McVay & Kane, 2009). Consistent with these findings, higher levels of brain regions were found to be involved in MW (Christoff et al., 2009; Kawagoe et al., 2019). On the other hand, we can assume that PR is mainly a perceptual phenomenon. In addition to its spontaneous/unintentional alternation characteristic, it has been reported that lower sensory areas are involved in PR as well as higher associative brain areas (Leopold & Logothetis, 1999; Sterzer et al., 2009). Furthermore, it was reported that differences in bottom-up neural processing can explain individual differences in the duration of PR (Megumi et al., 2015) and that the concertation of gamma-aminobutyric acid in the primary visual cortex plays a modulatory role in the occurrence of PR (van Loon et al., 2013).

Consistent with these previous findings, our current results suggest that higher cognitive and lower perceptual systems may independently underlie the occurrence of MW or PR. As for changes in pupil diameter associated with fluctuations in arousal/attentional state, the occurrence of MW was reported to be coupled with smaller pupil diameter (Grandchamp et al., 2014; Unsworth & Robison, 2016, 2018). In contrast, dilation of pupil diameter was reported to be associated with the occurrence of perceptual changes and later stability of perception in PR (Einhäuser et al., 2008). These contrasting relationships and characteristics, as well as our current findings, would imply the existence of independent contributions/functions for arousal/attentional processes in MW or PR. These ideas should be tested directly in the future by comparing the spatial and temporal aspects of brain activities related to the occurrence of MW/PR, as well as by measuring changes in pupil diameter. The current study estimated each participant's tendency for MW and PR independently in order to investigate the possible relationships between each of the processes underlying MW and PR. Specifically, we focused primarily on trait levels of MW. A future study should develop and use a common experimental task to measure PR and MW states simultaneously.

## Supplemental Material

sj-pdf-1-ipe-10.1177_20416695231214888 - Supplemental material for No relationships between frequencies of mind-wandering and perceptual rivalry Click here for additional data file. Supplemental material, sj-pdf-1-ipe-10.1177_20416695231214888 for No relationships between frequencies of mind-wandering and perceptual rivalry by Souta Hidaka, Miyu Takeshima and Toshikazu Kawagoe in i-Perception
