# Supplementary material for: No relationships between frequencies of mind-wandering and perceptual rivalry
Source: Iperception. 2023 Nov 27;14(6):20416695231214888. doi: 10.1177/20416695231214888 (PMC10683402; doi:10.1177/20416695231214888)
Supplement: sj-pdf-1-ipe-10.1177_20416695231214888 - Supplemental material for No relationships between frequencies of mind-wandering and perceptual rivalry [file sj-pdf-1-ipe-10.1177_20416695231214888.pdf]

Supplementary Information

No relationships between frequencies of mind wandering and perceptual rivalry

Souta Hidaka[1,2], Miyu Takeshima[1], and Toshikazu Kawagoe[1,3]

1. Department of Psychology, Rikkyo University, 1-2-26 Kitano, Niiza-shi, Saitama, 352-8558 Japan

2. Department of Psychology, Faculty of Human Sciences, Sophia University, 7-1 Kioi-cho, Chiyoda-ku, Tokyo 102-8554, Japan.

3. Department of Community and Social Studies, Tokai University, 9-1-1 Toroku, Kumamoto-shi Higashi-ku, Kumamoto, 862-8652 Japan

*Corresponding author:

Souta Hidaka

E-mail: hidaka@sophia.ac.jp

Address: Department of Psychology, Faculty of Human Sciences, Sophia University, 7-1 Kioi-cho, Chiyoda-ku, Tokyo 102-8554, Japan.

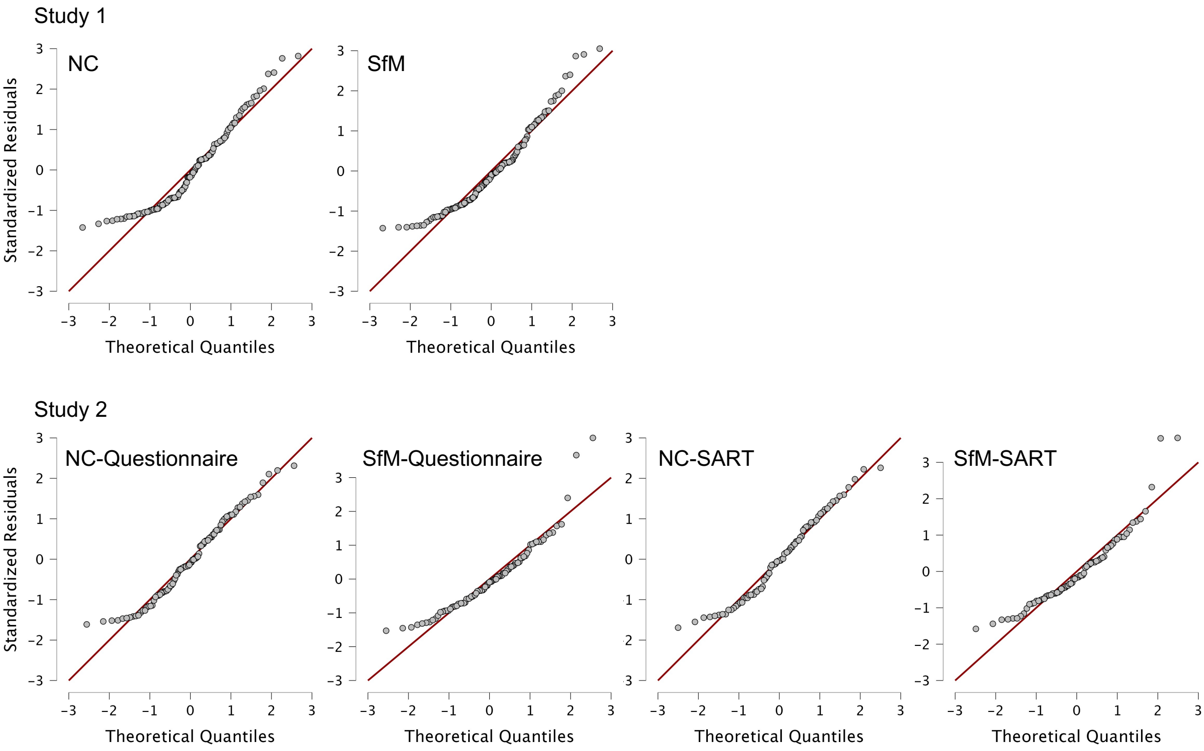

Supplementary figure S1. Q-Q plots for multiple regression analyses.

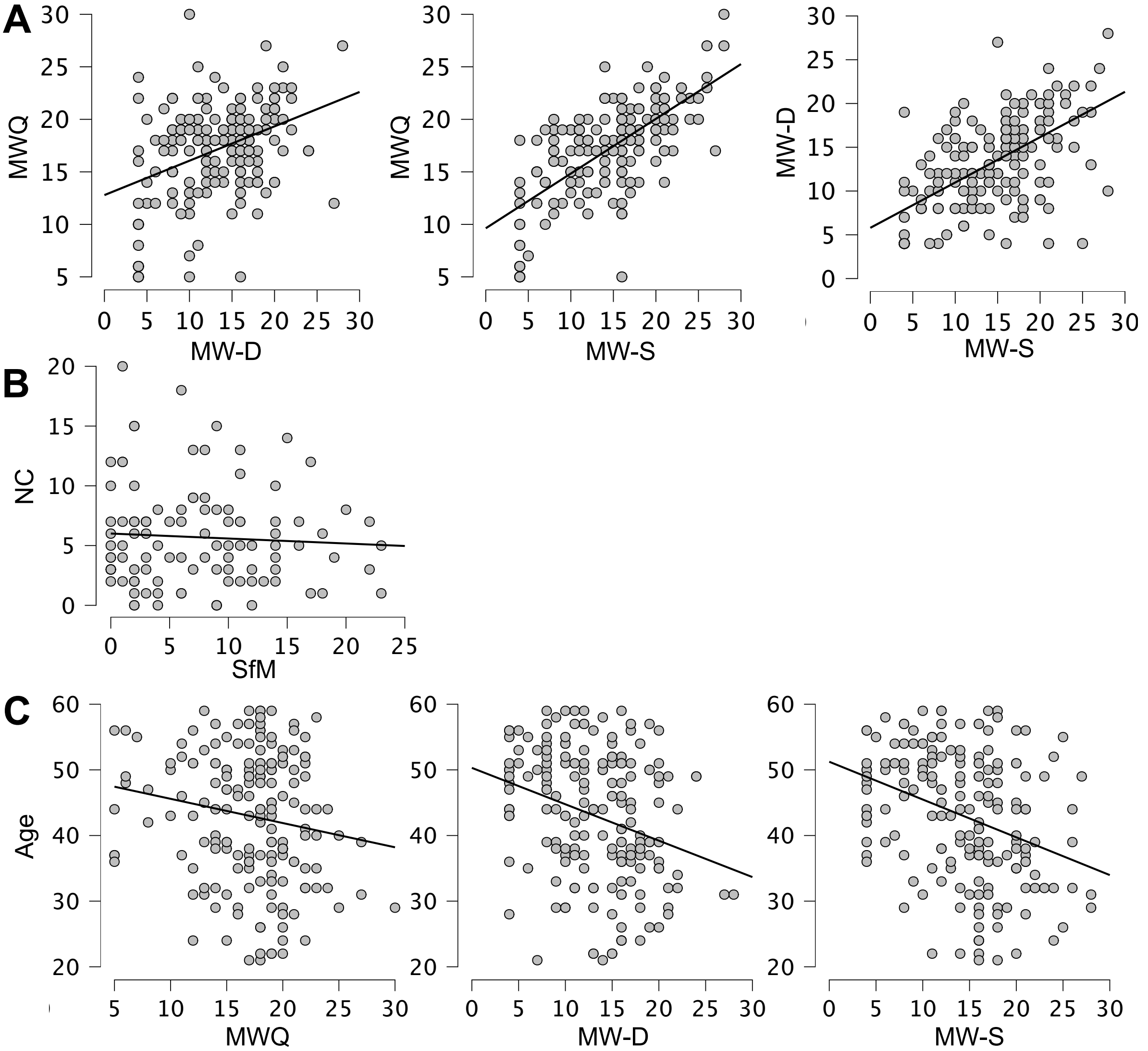

Supplementary figure S2. Plots for the results of Study 1. (A) Scatter plots among MWQ, MW-D, and MW-S scores. (B) A scatter plot between the numbers of PR between NC and SfM. (C) Scatter plots between age and MWQ, MW-D, or MW-S scores.

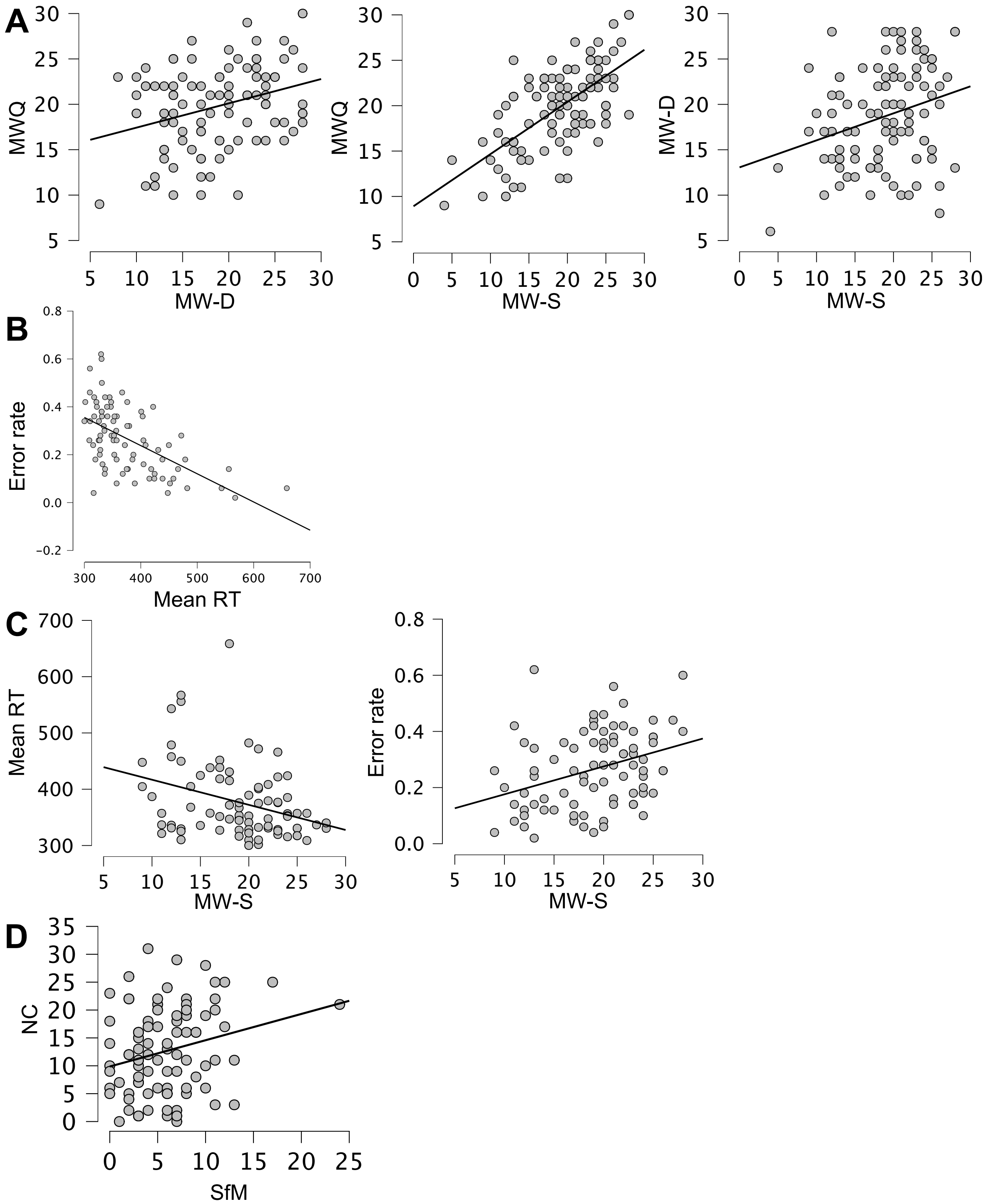

Supplementary figure S3. Plots for the results of Study 2. (A) Scatter plots among MWQ, MW-D, and MW-S scores. (B) A scatter plot between the error rates and the means of RT in SART. (C) Scatter plots between the MW-S scores and the mean RT or the error rates in SART. (D) A scatter plot between the numbers of PR between NC and SfM.
